# Genome Size Diversity in *Lilium* (Liliaceae) Is Correlated with Karyotype and Environmental Traits

**DOI:** 10.3389/fpls.2017.01303

**Published:** 2017-07-26

**Authors:** Yun-peng Du, Yu Bi, Ming-fang Zhang, Feng-ping Yang, Gui-xia Jia, Xiu-hai Zhang

**Affiliations:** ^1^Beijing Agro-Biotechnology Research Center, Beijing Academy of Agriculture and Forestry Sciences Beijing, China; ^2^Beijing Key Laboratory of Agricultural Genetic Resources and Biotechnology, Beijing Engineering Technology Research Center of Functional Floriculture Beijing, China; ^3^Beijing Key Laboratory of Ornamental Plants Germplasm Innovation & Molecular Breeding, National Engineering Research Center for Floriculture and College of Landscape Architecture, Beijing Forestry University Beijing, China

**Keywords:** karyotype, environmental traits, DNA content, genome size evolution, *Lilium*, phylogeny, adaptation

## Abstract

Genome size (GS) diversity is of fundamental biological importance. The occurrence of giant genomes in angiosperms is restricted to just a few lineages in the analyzed genome size of plant species so far. It is still an open question whether GS diversity is shaped by neutral or natural selection. The genus *Lilium*, with giant genomes, is phylogenetically and horticulturally important and is distributed throughout the northern hemisphere. GS diversity in *Lilium* and the underlying evolutionary mechanisms are poorly understood. We performed a comprehensive study involving phylogenetically independent analysis on 71 species to explore the diversity and evolution of GS and its correlation with karyological and environmental traits within *Lilium* (including *Nomocharis*). The strong phylogenetic signal detected for GS in the genus provides evidence consistent with that the repetitive DNA may be the primary contributors to the GS diversity, while the significant positive relationships detected between GS and the haploid chromosome length (HCL) provide insights into patterns of genome evolution. The relationships between GS and karyotypes indicate that ancestral karyotypes of *Lilium* are likely to have exhibited small genomes, low diversity in centromeric index (CV_CI_) values and relatively high relative variation in chromosome length (CV_CL_) values. Significant relationships identified between GS and annual temperature and between GS and annual precipitation suggest that adaptation to habitat strongly influences GS diversity. We conclude that GS in *Lilium* is shaped by both neutral (genetic drift) and adaptive evolution. These findings will have important consequences for understanding the evolution of giant plant genomes, and exploring the role of repetitive DNA fraction and chromosome changes in a plant group with large genomes and conservation of chromosome number.

## Introduction

In general, >2,000-fold diversity in genome size (GS) is observed among plants (Kelly et al., 2015), and GS may differ by >40-fold among species of the same ploidy within a single genus of plants (Pellicer et al., 2010; Kelly et al., 2015). The diversity of GS is of fundamental biological importance and has been a longstanding puzzle in evolutionary biology (Bennett and Leitch, 2005, 2011). The existing diversity is not restricted to differences between species, as extensive GS diversity also exists within species (Díez et al., 2013; Long et al., 2013; Ågren et al., 2015). Elucidation of the evolutionary processes underlying this diversity has received much attention (Petrov, 2001; Gaut and Ross-Ibarra, 2008; Lynch, 2011; Ågren and Wright, 2011). Such wide diversity has been hypothesized to be the result of several genetic mechanisms. Neutral (GS is assumed to evolve until the loss of DNA is maintained equally to the rate of DNA gain; Petrov, 2002), maladaptive (The restructuring of eukaryotic genomes was initiated by nonadaptive processes; Lynch and Conery, 2003) and adaptive (GS evolves as an adaptation to stressful environments) models (Gregory, 2002) have been proposed to explain GS diversity, yet there is little consensus about these processes.

In the absence of polyploidy, changes in the amount of repetitive DNA (transposable elements and tandem repeats) are primarily responsible for GS differences between species. As reported in *Fritillaria*, the result indicate that a lack of deletion and low turnover of repetitive DNA are major contributors to the evolution of extremely large genomes (Kelly et al., 2015). However, traits are the result of a combination of genotype and environment. Both neutral and selective evolutionary processes may influence GS diversity. Several studies have indicated that GS may evolve neutrally, with increases and decreases mainly being attributed to biases in insertion and deletion rates or recombination rates (Oliver et al., 2007; Nam and Ellegren, 2012; Ågren et al., 2015). GS may also correlate with ecologically or cytologically relevant traits or phenotypes, including latitude, altitude, temperature, precipitation, chromosome size, flowering time, flower size, leaf size and photosynthetic rates (Beaulieu et al., 2007a, 2008; Weng et al., 2012; Díez et al., 2013; Kang et al., 2014; Jordan et al., 2015). GS is positively correlated with total karyotype length and mean chromosome length in *Echinops* and related genera (Asteraceae, Cardueae) (Garnatje et al., 2004), suggesting an association between these parameters, as indicated by several authors, such as Dimitrova and Greilhuber (2000) for *Crepis* and Torrell and Vallès (2001) for *Artemisia*.

However, the conclusions drawn in various studies about correlated evolution between GS and ecological factors have often produced conflicting results. For example, a significant positive correlation between GS and altitude has been observed in *Zea mays* (Rayburn and Auger, 1990); however, the GS diversity of phylogenetically independent maize lineages is negatively correlated with altitude (Díez et al., 2013). Analyses of tribe Cardueae and tribe Anthemideae (Asteraceae) indicated that GS is correlated with karyological, physiological and environmental characteristics (Garcia et al., 2004; Garnatje et al., 2004). Nevertheless, Jakob et al. (2004) found that at the higher taxonomical level of *Hordeum* species (Poaceae), environmental correlations were absent. This lack of correlation could be attributed to the superimposition of life-form changes and phylogenetic constraints, which conceal ecogeographical correlations. Thus, the questions arise of whether the relationship between GS and the environment is different in different plants and what impact the methods applied for analysis and the sampling of species have on the obtained results.

Recent studies have indicated that ecological factors probably play a more important role in shaping GS diversity at lower taxonomic levels than at higher levels (Jakob et al., 2004; Dušková et al., 2010; Kang et al., 2014). Hawkins et al. (2008) suggested that analyses among closely related species within a single genus should provide greater interpretive power than analyses comparing more distant lineages at higher taxonomic levels. Unfortunately, studies addressing GS diversity among closely related species and its relationship with phenotypes as well as karyological and ecological factors are still scarce (Šmarda et al., 2007; Díez et al., 2013; Kang et al., 2014; Jordan et al., 2015).

As one of the most important biodiversity characteristics within the plant kingdom, GS has perhaps been best studied in the context of a single genus. As reported in *Allium* subgenus *Melanocrommyum*, particular species offer interesting cases for studying adaptive evolution, as many exhibit extensive geographical distributions across large spatial areas, landscapes or environmental conditions (Gurushidze et al., 2012). Within the range of a widespread species, individuals are likely to occur in diverse habitats along pronounced environmental gradients (Frenne et al., 2013; Lasky et al., 2013). The genus *Lilium* in the family Liliaceae (monocotyledons) is a phylogenetically important genus including approximately 110 species, which are distributed throughout cold and temperate regions of the northern hemisphere (Liang and Tamura, 2000). Fifty-five *Lilium* species are found in China (Liang and Tamura, 2000). De Jong (1974) and Patterson and Givnish (2002) described southwest China and the Himalayas as the point of origin of the genus *Lilium*. All species of the *Lilium* genus are diploid (*2n* = *2x* = 24) (Stewart, 1947) with the exception of *L. lancifolium*, which can also occur as a triploid (*2n* = *3x* = 36) (Noda, 1986). Many *Lilium* species, ornamental cultivars and hybrids are cultivated for their esthetic value. In addition, the flowers and bulbs of these plants are regularly consumed as both food and medicine in many parts of the world, particularly in Asia. At present, the “medicine food homology” value of *Lilium* plants is receiving considerable attention with respect to their great commercial prospects (Munafo and Gianfagna, 2015).

Although, the biological significance and diversity of the evolution of GS in plants has received considerable attention, genus-wide studies of correlated evolution between GS and karyological and environmental factors using phylogenetically controlled approaches have thus far been lacking in *Lilium*. Understanding the correlated evolution between GS and karyological and ecological factors within *Lilium* may be helpful for understanding the evolutionary mechanisms influencing GS in *Lilium* as well as GS diversity in general and among plants in particular.

We hope to contribute to a comprehensive understanding of GS evolution in *Lilium* by analyzing GS along with karyological and ecological data in multiple taxa within a phylogenetic framework. The aims of this study were (1) to examine the distribution of GS among taxa; (2) to investigate whether GS correlates with karyological and ecological characteristics, or GS evolution might be closely related to ecology and phenology, as suggested by previous studies; and (3) to discuss the potential evolutionary and ecological forces impacting GS. Additionally, we ask whether the diversity of GS is influenced by both neutral and selective evolutionary processes.

## Materials and methods

### Plant material

Table S1 lists the 81 taxa studied, which represent 74 species and 7 varieties, including six *Nomocharis* species, *Notholirion bulbuliferum*, and *Cardiocrinum giganteum*. Karyotype, GS, and ecological data are also presented (Table S2). A total of 73 speccies were used for GS analysis. These species were selected to represent the phylogenetic, karyological, ecological and morphological range of the genus. Our collection of 36 taxa representing 25 species from four sections of the genus including *C. giganteum* distributed in China that we evaluated via flow cytometry was also included in the database (Table S3).

Living plants or seeds were collected in the field throughout the geographic range of the genus in China and grown in glasshouses at the germplasm conservation center at the Beijing Academy of Agriculture and Forestry Sciences.

### Karyotype analysis

Mitotic chromosomes were examined in root tips 0.5–1.0 cm long obtained from plantlets or stem root of the 20 taxa. Roots were pretreated in saturated *p*-dichlorobenzene in water for 4–6 h at room temperature, fixed in Carnoy's Fluid (ethanol: chlorophorm: acetic acid = 6:3:1, v: v: v) for at least 1 day, and stored in 70% ethanol at 4 ± 2°C for further studies. Then the root tips were macerated in 1N HCl for 8~10 min at 60°C, stained by 1% Carbol Fuchsin for about 10 min, and squashed on a glass slide.

Chromosome preparation and fluorescence *in situ* hybridization (FISH) were referred to our previous study (Du et al., 2014). Sequences were probed using the DNA clone, pTa71, which contains the 9-kb EcoRI fragment from the 45S ribosomal DNA of wheat. FISH was performed as described in a previous study (Barba-Gonzalez et al., 2005). The FISH idiogram was established by Image-Pro Plus 6.0 (Media Cybernetics, USA) and EXCEL2010. In the karyotype idiogram, chromosome arrangement was in accordance with the short arm carried from long to short (Stewart, 1947). Figure S1 shows an example of the FISH idiogram and associated karyotype idiogram of *L. henryi* and *L. rosthornii*. There were three to five individuals were studied, and the chromosomes of at least 5 metaphase plates from each individual were studied for counting chromosome numbers. Number, size and shape of chromosomes were observed, and karyotypic asymmetry was evaluated.

In order to compare the data obtained with the techniques here described, morphometric information for mitotic chromosomes was taken from previous works (in Table S1). A data matrix of karyotype features was built, including the chromosome HCL, karyotype asymmetry index (AsK%), CVs (CV_CI_ and CV_CL_), basic chromosome number (*x*), ploidy, and chromosome number (*2n*) (Table S1). AsK% was calculated as the ratio of the sum of the lengths of the long arms of individual chromosomes to the HCL of the chromosome complement (Arano, 1963). The CV_CI_ index is used to evaluate differences in centromere position for each chromosome in the karyotype and provides a measure of intrachromosomal asymmetry. In contrast, CV_CL_ provides a measure of interchromosomal asymmetry, as it reflects the variability of chromosome sizes within the karyotype. The karyotype asymmetry index analysis method was described previously by Paszko (2006).

AsK% = Length of long arms in chromosome set/Total chromosome length in set × 100%.

### Environmental data

For our collection site of each species, we recorded geographical data and altitude. For the rest, we refer to the relevant literature records. Point distribution records were obtained from the Global Biodiversity Information Facility (http://www.gbif.org/) and National Meteorological & Hydrological Services (NMHSs) worldwide (http://worldweather.wmo.int/en/home.html). These collected data was used to obtain the climatic information including distribution region, temperature and precipitation from the WoldClim 1.4 (5 min) generic grid format (Hijmans et al., 2005). These bioclimatic variables are a summary of the mean temperature and precipitation, which can describe the biological climate of a typical distribution area of a species. (Table S2).

### Genome size estimation

Nuclei were prepared by chopping 50–100 mg of fresh young leaves of the 25 species for which living material was available in modified extraction buffer (CyStain PI Absolute P, Partec, Swedesboro, NJ, USA) according to the protocol of Weng et al. (2012). The extraction buffer provided in the reagent kit was supplemented with 2% polyvinylpyrrolidone (PVP; Sigma, St. Louis, MO, USA) and 1% beta-mercaptoethanol (Sigma). Each leaf sample was chopped with a razor blade in a 60 mm Petrie dish on ice and allowed to incubate for approximately 3 min in the buffer on ice. The homogenate was transferred to a 30-μm CellTrics nylon mesh filter (Partec) and the nuclei suspension was collected in a 5 mL tube on ice. Nuclei suspensions for all samples were stained simultaneously with propidium iodide solution prepared according to the CyStain PI Absolute P (Partec) manufacturer's protocol with the addition of 2% PVP. After at least 30 min of incubation in the dark on ice, the GS of each species was determined using a FACSCalibur flow cytometer (Becton Dickinson, San Jose', CA, USA) equipped with the analysis program CellQuest. *Triticum aestivum* L. “Chinese Spring” was used as an internal standard (2C = 30.9 pg, 43.7% GC) (Marie and Brown, 1993). To estimate the GS of the investigated taxa, at least five individuals per species were analyzed. The analysis was repeated twice for each sample. A DNA content of 5,000–10,000 stained nuclei was determined for each sample. Base on the peak of internal standard and *Lilium* species, experimental GS were calculated following equation (Figure S2): 2C = (sample G1 peak mean/standard G1 peak mean) × standard 2C genome size (pg DNA).

The rest of the data were obtained from the Plant DNA C-values Database (http://data.kew.org/cvalues/) and previous studies (Van Tuyl and Boon, 1997; Siljak-Yakovlev et al., 2003; Muratović et al., 2005, 2010; Peruzzi et al., 2009).

### Phylogenetic analyses

**Figure 1:**
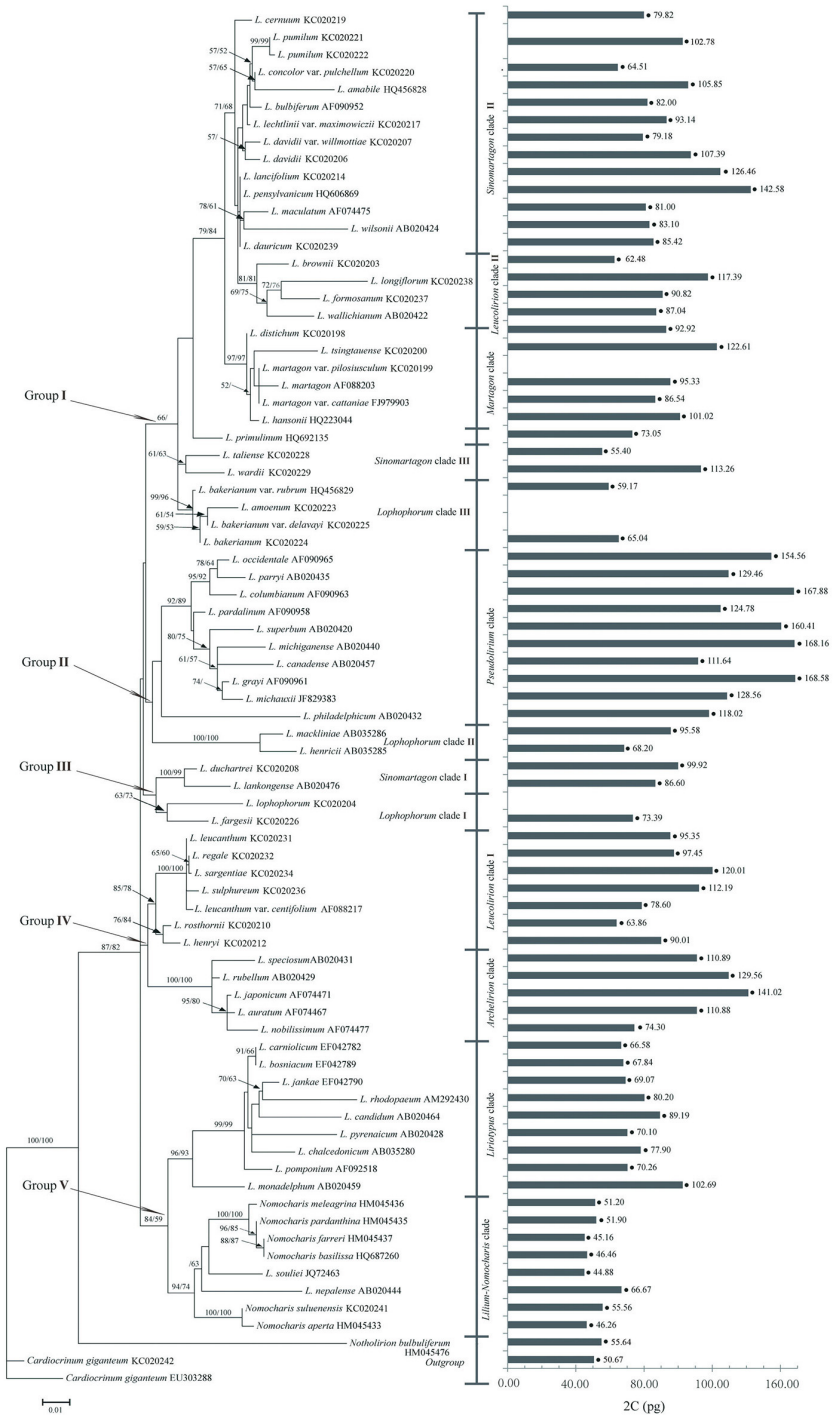
Molecular phylogeny of *Lilium* used in the PGLS analysis. Genome size mapped on the phylogenetic tree of 71 species of *Lilium* (including *Nomocharis*). *Nothorlirion bulbuiferum* and *Cardiocrinum giganteum* are outgroups. Phylogram of the 50% major consensus tree resulting from the Maximum Likelihood and Maximum Parsimony analysis of ITS dataset. Values along branches represent bootstrap (BS) of ML and MP, respectively.

To control for statistical non-independence, we accounted for phylogeny of *Lilium* previously generated by Du et al. (2014) in our statistical analysis. All sequence data were derived from analysis of the nuclear rDNA internal transcribed spacer (ITS) region (Du et al., 2014). Phylogenetic analyses were conducted using MP and ML. An MP tree was constructed using PAUP^*^ 4.0b10 (Swofford, 2002). An ML phylogenetic analysis was performed using RAxML 7.0.4 with unique model parameters (Stamatakis, 2006). A general time-reversible model was applied with a discrete gamma distribution. Bootstrap pseudo replicates were performed 1,000 times using the fast bootstrapping option and the best scoring ML tree. Phylogenetic trees were visualized using Treeview (Page, 1996). The best scoring tree was visualized with FigTree 1.3.1 (http://tree.bio.ed.ac.uk/). Species of *Notholirion* and *Cardiocrinum* were used as outgroup (Figure 1).

We first employed Pagel's λ (Pagel, 1999) implemented in the phylosig function in the *phytools* package (Revell, 2012) of R version 3.0.2 (Team, 2013) to test whether the data exhibited a significant phylogenetic signal. This package (Ågren et al., 2015) assesses the significance of phylogenetic signals by performing a likelihood ratio test against the null hypothesis that λ = 0. Next, we performed PGLS (Butler and King, 2004) regression between GS and karyological and ecological data, using the *ape* (Paradis et al., 2004) and *geiger* (Harmon et al., 2008) packages in R. We carried out the PGLS tests under both neutral (Brownian motion) and stabilizing selection (Ornstein–Uhlenbeck) models of trait evolution. AIC was employed to determine which model best described the data.

Evaluation of the significance of differences between groups/sections was based on calculation of the ANOVA and Tukey-HSD tests.

## Results

### Diversity in genome size in *Lilium*

**Figure 2:**
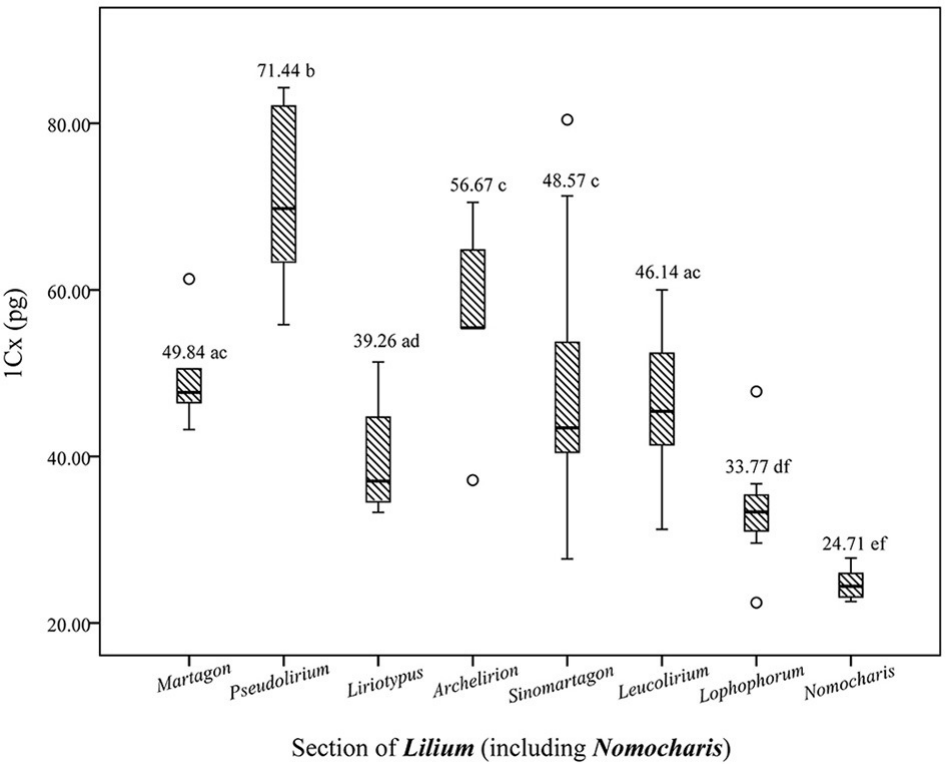
Boxplots illustrating the variability of genome size of different sections of *Lilium* (including *Nomocharis*). The outlined central box depicts the middle 50% of the data extending from the upper to lower quartile; the horizontal bar is at the median. The ends of the vertical lines indicate the minimum and maximum data values, unless outliers are present. Circles indicate outliers. Values with different letters are significantly different.

The evaluation of GS revealed considerable diversity among *Lilium* species. The GS estimates ranged from 44.88 pg, in *L. souliei*, to 167.58 pg, in *L. grayi* (Figure 1). The differences in GS observed between sections reflect considerable diversity. The largest component of GS diversity was attributed to Sinomartagon (Figure 2). Here, we describe the results in the order of the phylogenetic relationships within the genus. The phylogenetic tree was divided into five groups based on ITS sequences (Figure 1).

Group I consisted of *Martagon* clade, *Sinomartagon* clades II and III, *Leucolirion* clade II, *Lophophorum* clade III, and *L. primulinum*. The GS in this group ranged from 55.40 pg in *L. taliense* to 142.58 pg in *L. pensylvanicum*. Within Group I, *Martagon* clade had the highest GS, with the mean of the species ranging from 86.54 to 122.61 pg. *Sinomartagon* clade III had the lo west GS, ranging from 59.17 to 65.04 pg.

Group II consisted of *Pseudolirium* clade and *Lophophorum* clade II. GS between the diploid *Lilium* species differed by 2.47-fold, ranging from 68.20 pg in *L. henricii* to 168.58 pg in *L. grayi*. There was a significant difference in GS between *Pseudolirium* clade and *Lophophorum* clade II (81.89 vs. 143.21 pg, respectively).

Group III consisted of plants distributed in the Hengduan Mountains and the Himalayas: *Sinomartagon* clade I and *Lophophorum* clade I. GS in this group ranged from 73.39 pg in *L. fargesii* to 99.92 pg in *L. duchartri*. The DNA content of *Lophophorum* clade I (73.39 pg) was smaller than *Sinomartagon* clade I (93.26 pg).

Group IV contained plants distributed in Eastern Asia: *Leucolirion* clade I and *Archelirion* clade. Within *Leucolirion* clade I, GS ranged from 63.86 pg in *L. henryi* to 120.01 pg in *L. sargentiae*; it ranged from 74.30 pg in *L. nobilissimum* to 141.02 pg in *L. japonicum*. GS in *Leucolirion* clade I (93.92 pg) was slightly smaller than *Archelirion* clade (113.33 pg).

Group V consisted of *Lilium*-*Nomocharis* clade, which is distributed in the Himalayas, and *Liriotypus* clade, which includes European lilies. GS in this group ranged from 44.88 pg in *L. souliei* to 102.69 pg in *L. monadelphum*. A significant difference in GS was observed between *Lilium*-*Nomocharis* clade and *Liriotypus* clade (51.01 vs. 77.09 pg, respectively).

Analysis of variance (ANOVA) revealed significant differences in GS at the section level (Figure 2). In general, there are four main geographical distribution areas for the genus *Lilium*: the H-D Mountains and Himalayas; Eastern Asia; North America; and Europe-Western Asia. The H-D Mountains and Himalayas represent the origin and differentiation centers of the genus *Lilium* (Patterson and Givnish, 2002; Gao et al., 2013). North American species (sect. *Pseudolirium*) exhibit a relatively larger GS (1Cx = 71.44 pg), whereas the H-D Mountain and Himalaya (*Lilium-Nomocharis*) clades possess a smaller GS (1Cx = 38.26 pg) than the Europe-Western Asia (1Cx = 39.91) and Far East Asia clades (1Cx = 52.10 pg; Figure 2, Table 1).

**Table 1:** Comparison of means of DNA amount per basic chromosome set of *Lilium* in the four distribution regions.

**Geographical distribution**	**Mean 1Cx (pg)**	**Homogeneous groups**
Hengduan Mountains and Himalayas	38.26	a
Far East Asia	52.10	b
Europe and West Asia	39.91	a
North America	71.44	c

### PGLS test and analysis

Pagel's λ was used to detect the phylogenetic signal of GS across the examined *Lilium* species. Pagel's λ was found to be 0.61 (*P* < 0.001, λ > 0), indicating a significant phylogenetic signal in GS. Phylogenetic non-independence should therefore be taken into account in statistical analyses. Based on the indistinct relationship between geography (elevation, distribution region) and GS, we assumed that GS evolves under a neutral Brownian motion model (*df* = 71, *P* = 0.1794; *P* = 0.9642), moving toward a selective optimum in an Ornstein–Uhlenbeck model of stabilizing selection (*df* = 71, *P* < 0.0001; *P* = 0.9972). Akaike's information criterion (AIC) scores suggests that the Brownian motion model (AIC = −277.0011; −73.0787) describes the data better than the Ornstein–Uhlenbeck model (AIC = −98.1078; −68.7693).

### Relationship between GS and karyological characteristics

**Figure 3:**
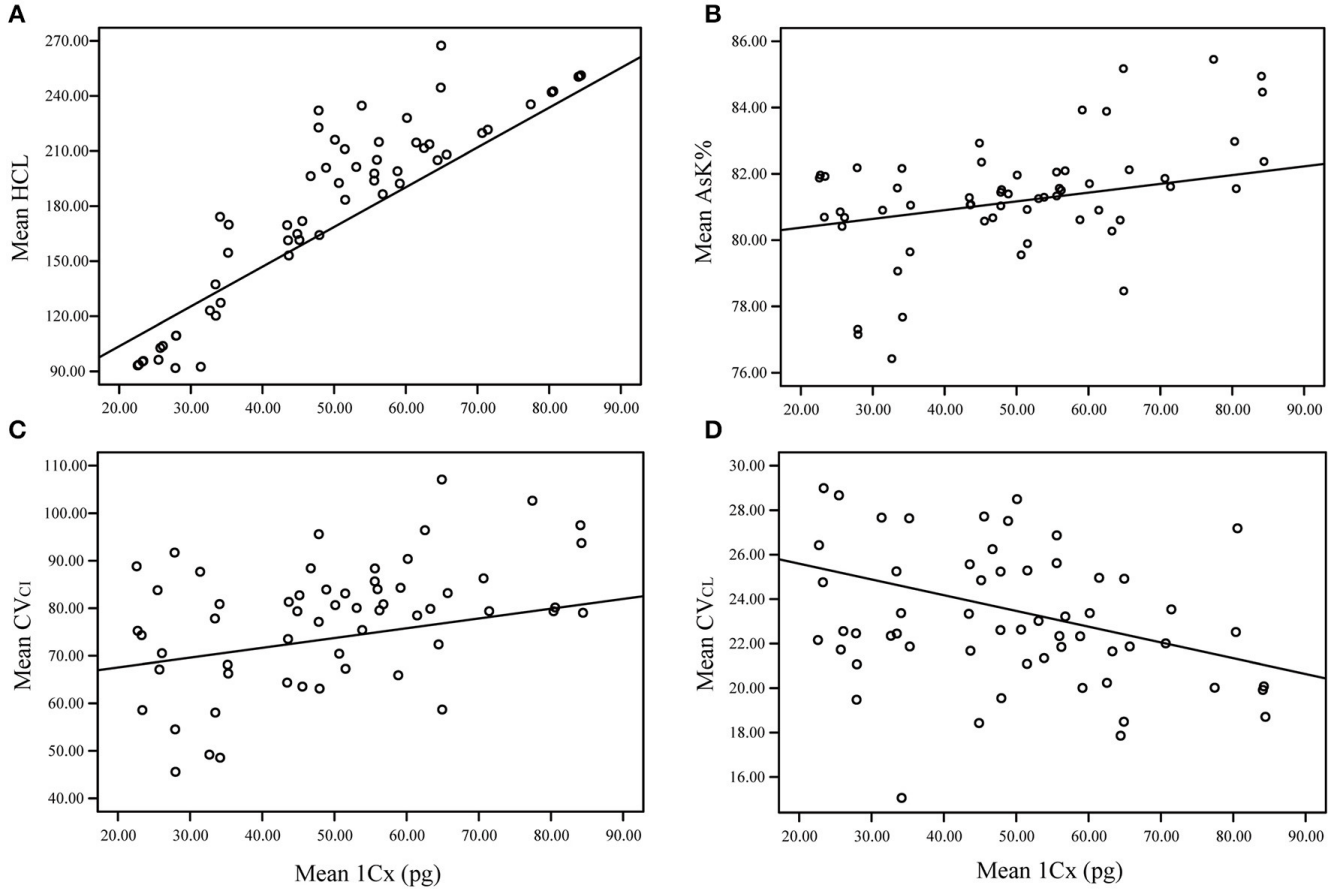
Relationship between genome size (1Cx) and karyotype features including chromosome total haploid length (HCL), karyotype asymmetry index (AsK%), coefficients of variation (CVs) (CV_CI_ and CV_CL_), based on the linear regression model inferred using phylogenetic generalized least squares (PGLS) in R. **(A)** chromosome total haploid length (HCL); **(B)** karyotype asymmetry index (AsK%); **(C)** diversity in centromeric index (CV_CI_); **(D)** variation in chromosome lengths (CV_CL_).

**Figure 4:**
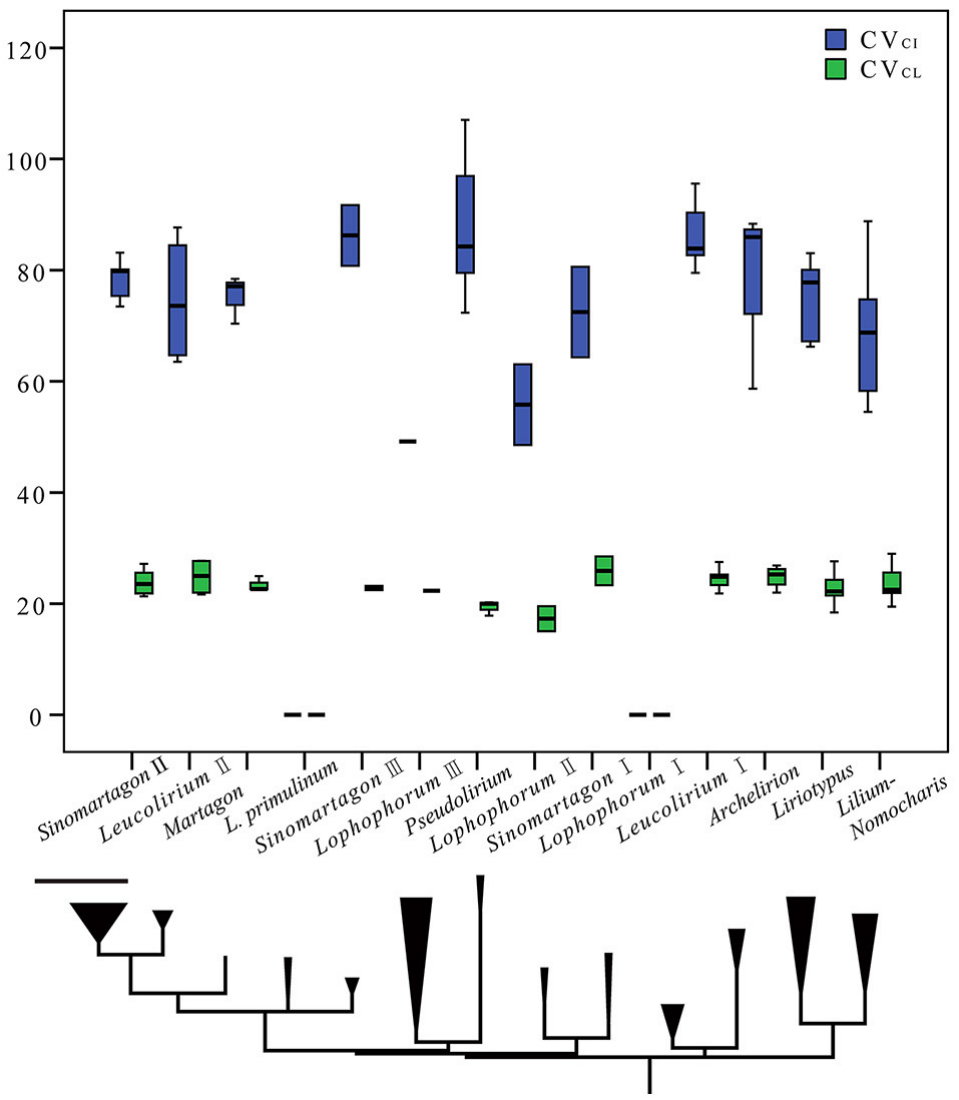
Clustered boxplots illustrating the variability of both the coefficient of variation (CV) of the centromeric index (CV_CI_) and chromosome length (CV_CL_). The outlined central box depicts the middle 50% of the data extending from the upper to lower quartile; the horizontal bar is at the median. The ends of the vertical lines indicate the minimum and maximum data values, unless outliers are present. Circles indicate outliers. Taxa are ordered by phylogenetic grouping (according to the phylogenetic tree on the bottom of the graph, taken from Figure 1).

According to Levin (2002), the correlation between HCL and 1Cx (monoploid GS) typically exceeds *r* = 0.85 both within species and between species of related genera. HCL has therefore been considered a suitable proxy for GS. Phylogenetic generalized least squares (PGLS) analyses showed a strong positive relationship between 1Cx and HCL (*P* < 0.001) in *Lilium* (Figure 3A). The pairwise correlations between 1Cx and AsK% were positive, suggesting a close association between the two parameters (*P* = 0.0078; Figure 3B). Two coefficients of diversity (CV) indices were found to be particularly informative in the measurement of asymmetry. A generally positive correlation was observed between 1Cx and (relative diversity in centromeric index) CV_CI_ across *Lilium* (*P* = 0.0283; Figure 3C), whereas (relative diversity in chromosome length) CV_CL_ and 1Cx showed a strong negative relationship in *Lilium* (*P* < 0.001; Figure 3D). Boxplots showing the range of values obtained for these two parameters for each clade arranged phylogenetically are presented in Figure 4. Based on the weights of the karyology coefficients, HCL, AsK%, and the CV indices could be considered suitable predictors of GS.

### Correlations between GS and geographic and bioclimatic factors

**Figure 5:**
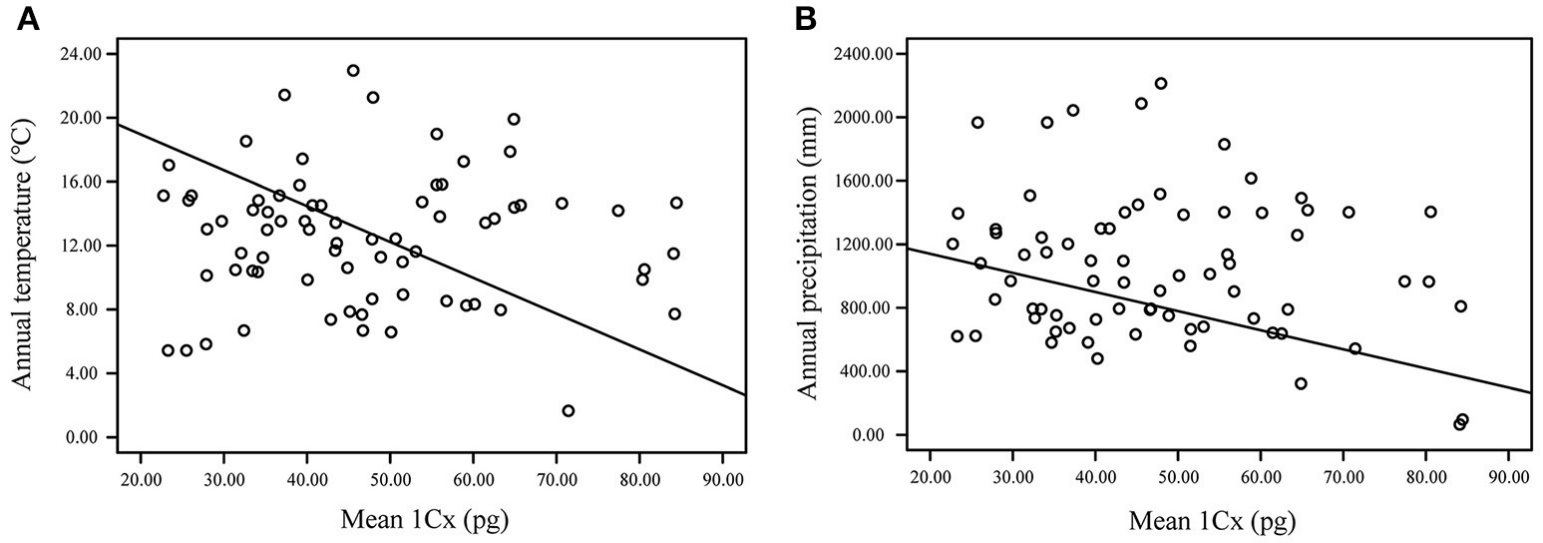
Relationship between genome size (1Cx) and environmental traits including annual average temperature and annual precipitation based on the linear regression model inferred using phylogenetic generalized least squares (PGLS) in R. **(A)** annual average temperature; **(B)** annual precipitation.

Geography (e.g., elevation, longitude and latitude) has been used as a proxy for a suite of environmental variables. In particular, the relationship between elevation and GS has long been of interest. Therefore, we asked whether this diversity follows a discernible pattern with environmental factors in *Lilium*. The relationship between 1Cx and elevation was found to be generally negative (*P* = 0.1794). In contrast, bioclimatic factors (annual temperature and precipitation) were strongly correlated with GS (*P* < 0.001, *P* < 0.001; Figure 5). Although, ANOVA analysis revealed significant differences in GS at the section level. There was an overlap-distribution between groups in different distribution areas. For example, species of sect. *Leucolirium* 6b are mainly distributed in East Asia, and sect. *Leucolirium* 6a species are mainly distributed in the regions of H-D Mountains and Himalayas. Therefore, the PGLS analysis showed that the correlation between GS and the distribution region is not clear.

Each coefficient provides insight into its importance in the model. The weights of the obtained coefficients indicated that the most highly weighted bioclimatic predictors for *Lilium* are related to temperature and precipitation.

## Discussion

We performed the first comprehensive study exploring the diversity and evolution of GS and its correlation with karyological, geographic and bioclimatic traits within the genus *Lilium*. Strong phylogenetic signals (λ = 0.61 for 1Cx) in GS were detected, indicating that GS is generally phylogenetically conserved among closely related species. The strong phylogenetic signals observed suggest that GS diversity in *Lilium* is probably a result of genetic drift or neutral evolution. The observation showed that transposable elements (TEs) abundance play an important role in governing the genome size diversity, such as in rice (Piegu et al., 2006), cotton (Hawkins et al., 2006), *Arabidopsis* (Hu et al., 2011), and *Fritillaria* (Kelly et al., 2015). The significant positive relationships detected between GS and HCL provided some insights into patterns of genome evolution, and HCL can also be used a proxy for GS across *Lilium*. Significant relationships between GS and karyotypes indicate that ancestral karyotypes of *Lilium* are likely to have exhibited small genomes, low CV_CI_ values, and relatively high CV_CL_ values. The significant relationships between GS and annual temperature and between GS and annual precipitation suggest that adaptation to habitat has strongly affected GS diversity. Our results document the flexibility in the size of the *Lilium* genome and provide strong evidence supporting an adaptive hypothesis of GS evolution in *Lilium*.

### Phylogenetic signal of GS

Several studies have shown strong phylogenetic dependence of GS at higher taxonomic scales (Beaulieu et al., 2007b; Knight et al., 2010; Whitney et al., 2010; Bainard et al., 2012; Kamilar and Cooper, 2013). However, only a few studies have quantified the strength of the phylogenetic signal for plant GS at the genus level. Strong phylogenetic signals for GS were detected in *Orobanche* (λ = 1) (Weiss-Schneeweiss et al., 2006), *Hieracium* (λ = 0.908) (Chrtek et al., 2009), *Filago* (λ = 0.934) (Andrés-Sánchez et al., 2013), and *Primulina* (λ = 0.939) (Kang et al., 2014). The strong phylogenetic signal (λ = 0.61 for 1Cx) found for GS, together with previous evidence, indicates that GS is generally phylogenetically conserved among closely related species. The strong phylogenetic signals detected for GS suggest that the GS diversity observed in *Lilium* is probably a result of neutral evolution (genetic drift).

### Correlated evolution of GS vs. karyotype

Changes in the morphological characteristics of chromosomes are believed to be related to evolution in higher plants (Zarco, 1986). Chromosome diversity may have important effects on the evolution of *Liliacea*, as such changes could substantially affect the fine structure of chromosomes and karyotype asymmetry (Peruzzi et al., 2009; Gao et al., 2012). Although the degree of accuracy of chromosome analyses depends on the method of measurement, the significant positive relationship between GS and HCL (*P* < 0.001) indicated broad agreement between the GS values obtained using GS estimation techniques and those inferred from HCL measurements (Figure 3A; Figure S1). Our results highlight the potential of using chromosome data as a proxy for GS, as noted in previous reports (Levin, 2002; Leitch et al., 2009; Peruzzi et al., 2009).

The positive correlation found between GS and AsK% in the present study (Figure 3B) indicates that additional DNA has been added on the long arm in *Lilium*. There was a strong positive correlation between 1Cx and CV_CI_, indicating that increases in GS were generally accompanied by increasing karyotype asymmetry through increasing the variability of centromere position (Figure 3C). In contrast, the strong negative relationship identified between 1Cx and CV_CL_ in *Lilium* indicates that increases in GS are generally accompanied by decreasing size differences between chromosomes in the karyotype (Figure 3D). The analysis of changes in karyotype asymmetry with GS provided some insight, indicating that additional DNA is mainly added to the long arms of smaller chromosomes, rather than being distributed uniformly across the karyotype. These results agree with those previously reported by Peruzzi et al. (2009) based on an analysis of subgroups within Liliaceae, including *Tricyrtis* and Lilioideae. In general, there is a positive correlation between GS and the percentage of repetitive DNA (TEs and tandem repeats) in the absence of polyploidy (Levin, 2002; Kelly et al., 2015).

However, there is ongoing debate about the relative importance of amplification versus deletion of repetitive DNA in governing GS. Based on the whole-genome sequences of three evening *primrose* species (*Oenothera*), Ågren et al. (2015) found that GS was not associated with TE abundance. Instead, the larger genomes exhibited a higher abundance of simple sequence repeats. Additionally, a lack of deletion and low turnover of repetitive DNA are major contributors to the evolution of extremely large genomes in *Fritillaria* (Kelly et al., 2015). As allied genera, the pattern of GS evolution found in *Lilium* might be similar to that of *Fritillaria*. However, these two genera show different, or even opposite patterns of karyotype evolution (Peruzzi et al., 2009; Gao et al., 2012). Therefore, future work should test the contribution of repetitive DNA to GS evolution across *Lilium*.

Divergence time dating and lineage sorting analyses of *Lilium* (Gao et al., 2013) showed a congruence trend with GS evolution, such that a small GS represents the ancestral state with other clades, and the predominant direction of GS evolution is upwards (Hawkins et al., 2008; Leitch et al., 2010). The significant correlation detected suggests that ancestral karyotypes of *Lilium* are likely to have exhibited small genomes, low CV_CI_ values, and relatively high CV_CL_ values (Figure 4). Our analysis showed that there was a general tendency of increases in GS during evolution. Furthermore, the H-D and Himalayan clades appear to be more susceptible to high rates of extinction than lineages in other areas. The existence of clades/species with low diversity (e.g., *L. duchartrei, L. lankongense*) within relatively old lineages may be explained by high extinction rates (Gao et al., 2013). These clades/species have adapted to the environment through speciation over time but are often narrowly distributed. From this evolutionary point of view, GS diversity is shaped by adaptation to the microenvironment. While convergent morphology may occur within divergent groups, such as *Nomocharis* and *Lophophorum*, due to adaptations developed in response to similar new habitats, from perspective of biogeographic history, GS diversity is adjusted by evolutionary adaptation to the macroenvironment.

### Correlated evolution of GS vs. environmental traits

A related question is whether GS diversity is predictably influenced by natural selection, which would suggest that ecological factors can constrain GS. The significant phylogenetically independent association between GS and environmental traits observed in *Lilium* (Figure 5), as expected, is consistent with broad patterns across land plants (Beaulieu et al., 2008; Veselý et al., 2012; Díez et al., 2013; Kang et al., 2014; Jordan et al., 2015). The most highly weighted ecological predictors in *Lilium* are related to annual temperature and precipitation. In fact, many of these factors are not independent but are interrelated and are directly affected by geographic position or elevation. For example, high elevations imply low temperatures. Thus, we should treat ecological factors as a whole when addressing the relationship between GS and ecological factors.

It has been reported that *Lilium* evolved in the H-D and Himalayan mountains approximately 13.6 Mya (Gao et al., 2013). The major clades of the genus then emerged approximately 6–8 Mya, followed by a burst of speciation approximately 4 Mya, accompanied by expanding habitats and migration. Analyses of speciation and extinction rates showed general stability through time. Extinction rates have remained approximately the same since the evolution of *Lilium*, and speciation rates have declined slightly, suggesting that diversification has somewhat diminished over time (Gao et al., 2013). Thus, based on the evolutionary history of *Lilium*, the maintenance of a large range generally presents challenges in terms of balancing adaptive evolution and maintaining of species persistence and integrity (Lexer et al., 2013). Our results indicated that H-D Mountain and Himalayan species, such as those belonging to sect. *Lophophorum* and *Nomocharis*, which usually exhibit a relatively small GS, generally grow above 3,000 m in relatively extreme environments. In contrast, Far East and North American species normally grow at lower altitudes, with relatively less harsh environments, and display a larger GS (Figure 2, Table 1). The H-D Mountain and Himalayan environment is generally characterized by high elevation and cold stress and is often linked to a short growing period and short plant height. Under such conditions, plant uptake of nitrogen and phosphorus may be restricted. Thus, our results support the hypothesis that a small GS evolves as an adaptation to stressful environments. As mentioned above, H-D Mountain and Himalayan clades appear to be more susceptible to high rates of extinction than lineages in other areas (Gao et al., 2013). We can speculate that restricted ecological tolerances may increase the probability of extinction by reducing population sizes. Moreover, it is likely that the rich diversity of phenotypic traits in *Lilium* may often mirror the co-ordination of traits with components of geography and climate, suggesting that the distribution of genetic or genomic diversity may follow similar patterns reflecting selective factors (Ingvarsson and Street, 2011; Eckert and Dyer, 2012; Lasky et al., 2013; McKown et al., 2014).

In summary, we have performed the first large-scale investigation of the roles of karyotype and environmental traits in GS evolution in *Lilium*, based on extensive sampling of 71 species (81 taxa), representing c. 65% of all *Lilium* species. These findings will have important consequences for understanding the content and evolution of plant genomes, especially for plants with extremely large genomes. In the future, it will be necessary to address the underlying mechanisms of correlated evolution between traits to clarify the evolutionary forces driving *Lilium* GS diversity.

## Author contributions

YD, GJ, and XZ conceived the experiments, YD, MZ, and GJ collected the samples, YD and MZ conducted the experiments, YD, YB, and FY analyzed the results, YD and YB wrote the manuscript.

### Conflict of interest statement

The authors declare that the research was conducted in the absence of any commercial or financial relationships that could be construed as a potential conflict of interest.
