# Supplementary material for: Genome Size Diversity in Lilium (Liliaceae) Is Correlated with Karyotype and Environmental Traits
Source: Front Plant Sci. 2017 Jul 26;8:1303. doi: 10.3389/fpls.2017.01303 (PMC5526928; doi:10.3389/fpls.2017.01303)
Supplement: Supplementary file 4 [file Image1.PDF]

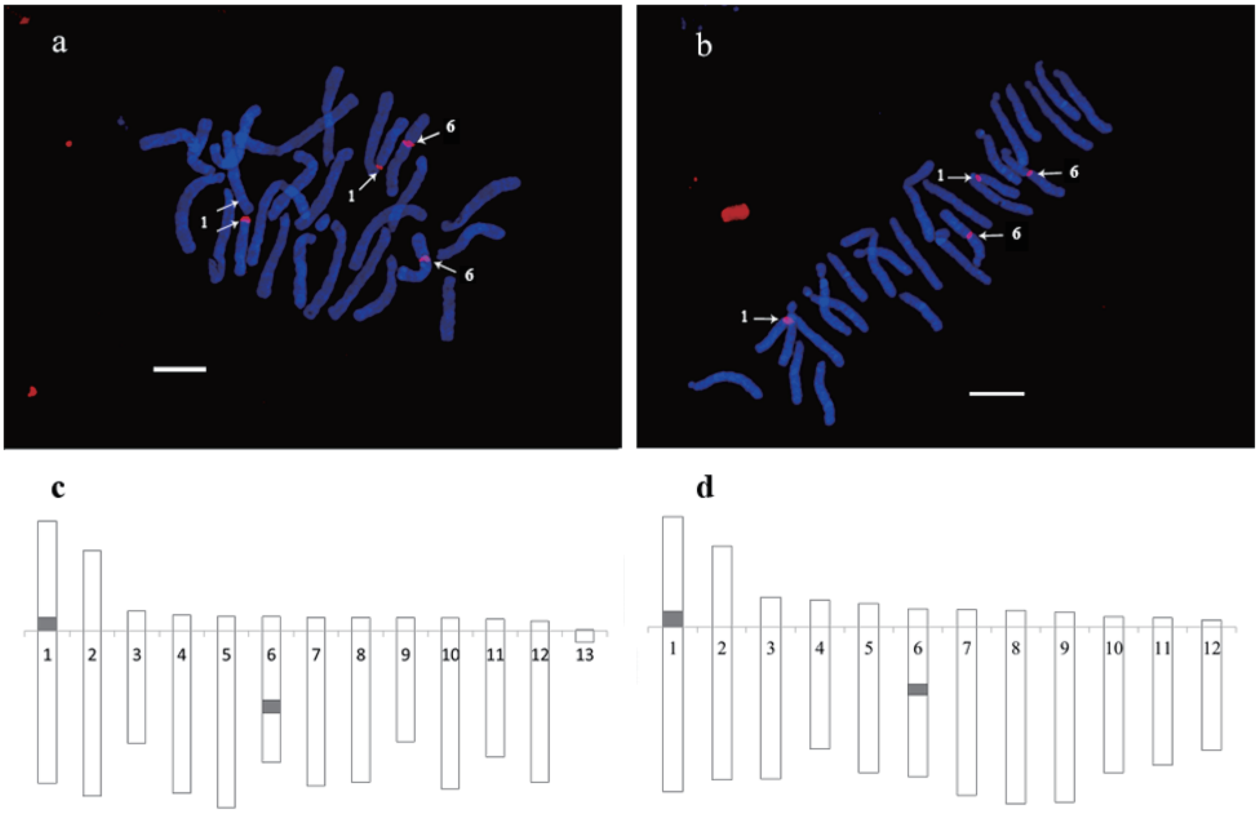

Figure S1. FISH analyses of *L. henryi* (KC020212) (a, c) and *L. rosthornii* (KC020211) (b, d) DAPI counterstaining (blue) and digoxigenin-labeled 45S rDNA probe (red indicates a 45S rRNA gene loci). Scale bar 10μm (Du et al., 2014)
