# Supplementary material for: Genome Size Diversity in Lilium (Liliaceae) Is Correlated with Karyotype and Environmental Traits
Source: Front Plant Sci. 2017 Jul 26;8:1303. doi: 10.3389/fpls.2017.01303 (PMC5526928; doi:10.3389/fpls.2017.01303)
Supplement: Supplementary file 5 [file Image2.PDF]

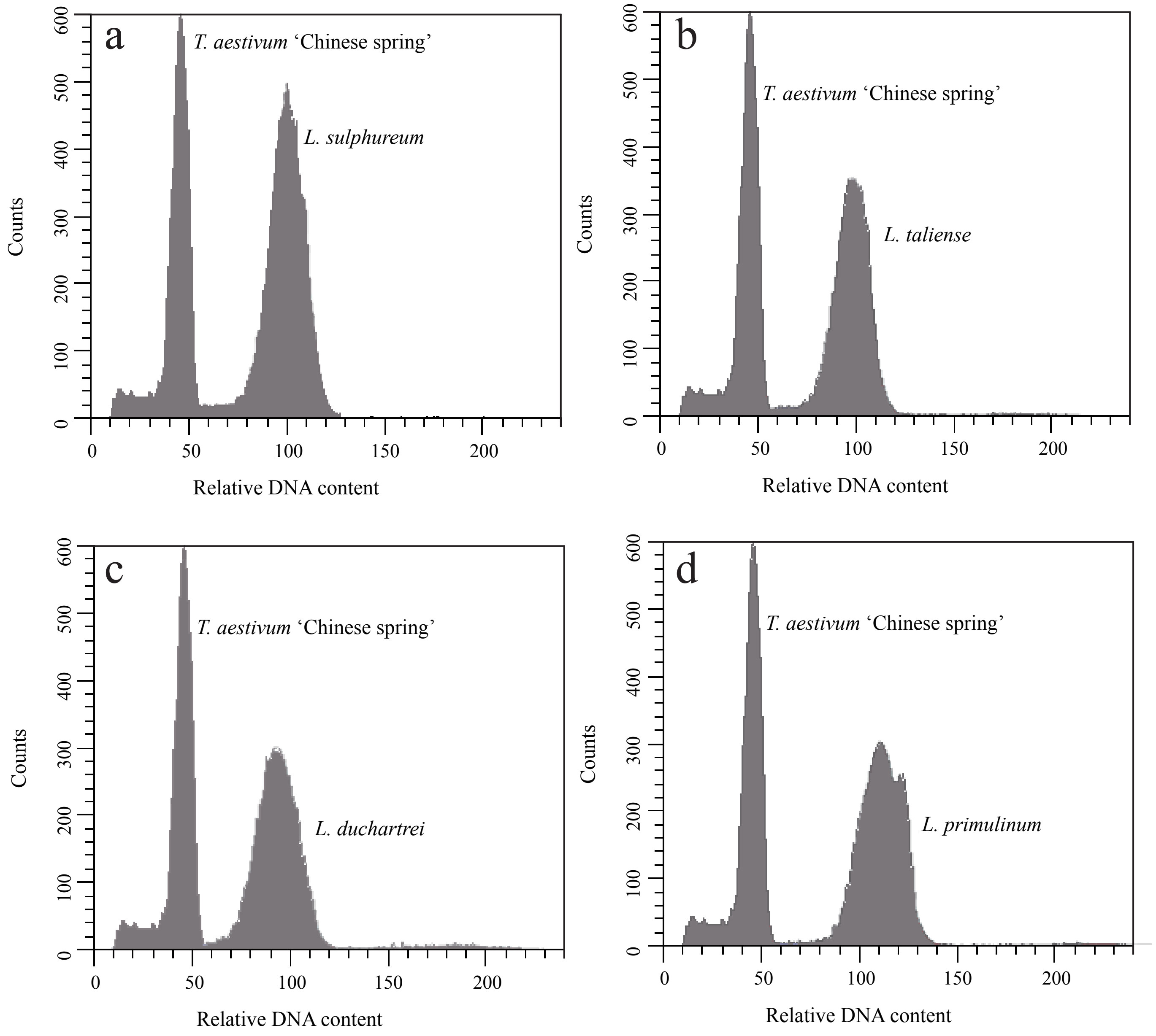

Figure S2. Histograms of the relative flow-cytometric genome size measurements in taxa of *Lilium*. Examples of relative fluorescence intensity obtained based on the analysis of propidium iodide-stained nuclei isolated from young leaves of the species of: (a) *L. sulphureum*, (b) *L. taliense*, (c) *L. duchartrei*, and (d) *L. primulinum* with the internal standard *Triticum aestivum* L. 'Chinese Spring'.
